# Emotional and Behavioural Problems of Children and Youth With Intellectual Disabilities Following the February 6 Kahramanmaraş Earthquake: A Mixed‐Methods Study

**DOI:** 10.1111/jar.70229

**Published:** 2026-04-19

**Authors:** Hatice Ulu Aydın, Emine Eratay

**Affiliations:** ^1^ Special Education Department Abant Izzet Baysal University Bolu Turkey

**Keywords:** children and youth, earthquake, emotional and behavioural disorders, intellectual disability, mixed‐methods, post‐disaster mental health

## Abstract

**Background:**

The February 6 Kahramanmaraş earthquake was associated with various emotional and behavioural difficulties observed in children with intellectual disabilities, who were exposed to the disaster either directly or indirectly. This study examined these emotional and behavioural outcomes among children and youth with intellectual disabilities in Türkiye.

**Methods:**

A mixed‐methods approach was employed. Quantitatively, teachers completed the ASEBA Teacher's Report Form for 52 participants, and data were analysed using Mann–Whitney *U* tests. Qualitatively, the House–Tree–Person test was administered to a subsample (*n* = 15) to explore emotional indicators.

**Results:**

Quantitative results showed significant social, attention, thought, and aggressive problems, with boys exhibiting higher levels of rule‐breaking and aggressive behaviours than girls. Qualitative findings confirmed emotional and behavioural difficulties.

**Conclusion:**

These findings reveal an urgent need for post‐earthquake psychosocial support and the development of needs‐based educational programs for children and youth with intellectual disabilities.

## Lay Summary

- This study indicates that children and youth with intellectual disabilities exhibited notable social, attention, and behavioral difficulties following the February 6 earthquake. Boys, in particular, showed higher levels of rule‐breaking and externally directed behaviors compared to girls.
- Projective drawing tests highlighted underlying emotional distress, with many participants depicting houses and trees lacking key details (e.g., chimneys or leaves), reflecting heightened anxiety and a sense of emptiness associated with the disaster experience.
- Since individuals with intellectual disabilities often experience difficulties in verbally expressing adverse experiences, there is an urgent need for developmentally appropriate, evidence‐based interventions tailored to their cognitive, communicative, and emotional needs. Support programs should specifically address the externalised behaviors seen in boys and provide non‐verbal therapeutic outlets.
- By combining teacher reports with drawing analyses, this research provides a vital model for assessing vulnerable populations in crisis zones, urging the global community to develop more inclusive disaster‐response strategies.

## 1 Introduction

### 1.1 Psychosocial Effects of Earthquakes on Children and Youth

Earthquakes, particularly those with a magnitude of 7.0 or higher on the Richter scale, can cause severe destruction, put many lives at risk, and adversely affect individuals' physical, psychological, and social development (Kaplan, Alkasaby, et al. 2024; Kaplan, Düken, et al. 2024; Ren et al. 2014; Stein and Stein 2022; Yoosefi Lebni et al. 2020). Türkiye is a country with high disaster risk and is among the most disaster‐prone countries in the world (Arslanlı et al. 2024). On 6 February 2023, at 04:17 and 13:24 Turkish time, two major earthquakes of 7.7 and 7.6 occurred in the Pazarcık and Elbistan districts of Kahramanmaraş province. Official sources report that 50,783 people lost their lives, 115,353 people were injured, and 37,984 buildings were destroyed as a result of the earthquakes (Disaster and Emergency Management Presidency [DEMP] 2023). The earthquakes affected an area of 108,812 km^2^ covering 11 provinces of Türkiye. These earthquakes are characterised as the disaster of the century for Türkiye and are recorded as the most devastating earthquakes in the country's history. It also negatively affected many people's economic and social lives (DEMP 2023). As a result of the destruction caused by the earthquake, many people were forced to live in containers or tents. These harsh conditions may be associated with various mental and physical health problems (Eroğlu and Yakşi 2023).

It is reported that more than 100 million children worldwide are affected by disasters each year (Schonfeld and Demaria 2015; United Nations Office for Disaster Risk Reduction 2011). Disasters that lead to widespread destruction and loss at the community level are considered among the potentially traumatic life events for children (National Traumatic Stress Network [NTSN], n.d.). Research highlights that children and adolescents are particularly vulnerable to the psychological ramifications of earthquakes. Düken, Küçükoğlu, and Kılıçaslan (2024) and Düken, Kaplan, and Almazan (2024) emphasised the prevalence of post‐traumatic stress and depressive symptoms in children following significant seismic events, such as those experienced during the Kahramanmaraş earthquake. Similar findings were reported by Goenjian (1993), who noted heightened post‐traumatic stress disorder (PTSD) symptoms among adolescents in Armenia after the Spitak earthquake, underscoring the profound emotional toll such disasters can take on young individuals. Furthermore, Giannakopoulos et al. (2025) elucidated the extended emotional impact that such earthquakes can have, often instilling a long‐lasting fear of future disasters in youth. In a study conducted in Wenchuan, China, Wang et al. (2012) found that even adolescents residing far from the epicentre exhibited notable rates of PTSD and depression, indicating that the influence of such traumatic events can extend well beyond those in immediate proximity. Such findings underline the critical necessity of understanding the broader effects of trauma across geographic and demographic boundaries.

Beyond these direct psychological effects, earthquakes can also give rise to secondary or indirect traumas. Individuals who were not directly exposed to the disaster may still experience significant emotional and behavioural consequences as a result of witnessing suffering, coping with chaos, or dealing with post‐disaster challenges (Demir and Namli 2024). Secondary‐level traumas arise from indirect exposure to traumatic events, affecting not only those who were present but also individuals who were indirectly impacted (Robinson‐Keilig 2014; Mancini 2019). For example, relatives of disaster victims may experience stress and anxiety disorders, reflecting the emotional upheaval they undergo in response to their loved ones' trauma. Similarly, individuals who follow disaster news through the media may also suffer psychological effects (Li et al. 2020). The psychological effects of secondary trauma are particularly pronounced in vulnerable groups such as children and youth, manifesting in emotional and behavioural problems such as anxiety, avoidance, altered perception of reality, sleep and appetite disturbances, physical complaints, frequent crying, restlessness, irritability, and unresponsiveness (Ali et al. 2012; Bal and Jensen 2007). Additional symptoms include sadness, guilt, attention and memory problems, nightmares, and decline in acquired skills (Yılancıoğlu and Özbaran 2023). The children and youth with intellectual disabilities (ID) in this study represent a group that was indirectly exposed (e.g., through media or household discussions) to the adverse effects of the earthquake; this may potentially contribute to secondary trauma, although these exposures were not directly measured in the study.

### 1.2 Disaster‐Related Vulnerabilities of Children and Youth With Intellectual Disabilities

Numerous studies have shown that, following disasters, children exhibit behavioural reactions such as crying and sleep disturbances, as well as emotional responses including sadness and grief (Myers 1994; Salloum and Overstreet 2008; Schonfeld and Demaria 2015). However, despite these findings, studies focusing on the emotional and behavioural problems experienced by children with intellectual disabilities in the context of disasters remain quite limited. Studies conducted on children with disabilities also indicated behavioural problems (Ducy and Stough 2021; Kimura 2020) and regression in social interaction skills (Mehtar and Mukaddes 2011; Valenti et al. 2012) after earthquakes or other traumatic events.

Peek and Stough (2010) conceptualised the exposure of children with disabilities to disasters across three dimensions: physical, psychological, and educational vulnerability, and noted that factors such as high levels of poverty, traumatic losses, and limited social support can further intensify these vulnerabilities. Within this framework, it is emphasised that each child with a disability may be affected by disasters in different ways and to varying degrees, with the process being shaped by multiple individual and environmental factors (Jang and Ha 2021). Importantly, individuals with intellectual disabilities represent a heterogeneous group (Hubert et al. 2019; Saleha et al. 2017), and disaster experiences may vary considerably depending on the level of support needs (Stough 2015). While children with lower support needs may experience anxiety and behavioral difficulties due to displacement, school disruption, and loss of routine, those with higher support needs (e.g., children with profound intellectual and multiple disabilities) may be at greater risk due to increased dependence on caregivers, communication challenges, and disruptions in specialised services (Boulet et al. 2009; Vohra et al. 2013).

Although disasters affect all segments of society, certain groups such as children, youth, and individuals with disabilities are exposed to disproportionately higher levels of risk. In this context, vulnerability refers to increased susceptibility to the adverse impacts of disasters resulting from the convergence of developmental characteristics, limited coping capacities, difficulties in accessing resources, and high dependence on caregivers and support systems (Fothergill 2017; Jones et al. 2020; Mathias et al. 2020; UNESCAP 2017). Disasters can have highly devastating consequences for these groups, including disruptions to daily routines, setbacks in academic achievement, and impairments in social development. Moreover, by elevating stress levels, disasters can also negatively affect children's overall health (Delicado et al. 2017; Peek and Stough 2010).

This multidimensional vulnerability framework provides an important basis for understanding why the psychological and behavioural effects of disasters may be more severe and long‐lasting among children, particularly those with intellectual disabilities. In the aftermath of an earthquake, increased levels of vulnerability among children and adolescents have been reported, along with elevated anxiety and social isolation. Higher levels of emotional and behavioural difficulties have been reported among children and youth with intellectual disabilities in post‐disaster contexts. Individuals in this group may present with significant emotional distress and behavioural differences, which may be related to cognitive and adaptive characteristics associated with their disabilities (American Association on Intellectual and Developmental Disabilities [AAIDD] 2024; Baudewijns et al. 2018). When these psychological difficulties co‐occur with additional stressors such as perceived loss of safety and disruption of support systems, more severe outcomes may be observed, underscoring the need for specialised interventions that address their specific needs and promote resilience (Jung and Han 2023).

### 1.3 Research on Disability and Intellectual Disability

Previous studies have investigated various aspects of disasters in relation to individuals with disabilities. Some studies focused on disaster preparedness and the teaching of disaster‐specific skills (Dixon et al. 2010; Gershon et al. 2013; Howard et al. 2017; Simpson et al. 2021; Ton et al. 2020; Wolf‐Fordham et al. 2015; Ulaşman and Sivrikaya 2025), while others examined the impacts of disasters on this population, including physical injuries, building collapses, and human loss (Fox et al. 2010; Good et al. 2016; Mehtar and Mukaddes 2011; Stough et al. 2016; Valenti et al. 2012). Teacher perspectives and school conditions during disasters have also been investigated (Ayyıldız et al. 2024; Boon et al. 2011; Chen et al. 2022; Ducy and Stough 2011; Nikolaraizi et al. 2021; Ronoh 2017; Söğüt and Kaya 2024; Stough et al. 2020). Additionally, research has documented the experiences of individuals with physical, hearing, and visual disabilities during disasters (Calgaro et al. 2021; Elisala et al. 2020; Finkelstein and Finkelstein 2020; Gartrell et al. 2020; Good et al. 2016; Pakjouei et al. 2021; Park et al. 2019; Stough et al. 2016; Yarımkaya et al. 2024).

For individuals with ID, research has focused on evacuation behaviours (Knudson et al. 2009; Shields et al. 1999) and instruction of disaster‐specific skills (Dixon et al. 2010). ID populations often face difficulties in recognising and responding to emergencies (Stough 2015). It is also important to note that the impacts of disasters can vary depending on the type of event and associated consequences (Jang and Ha 2021). Despite the growing number of studies, no research has yet directly examined the emotional and behavioural problems of individuals with ID following disasters. Some studies have explored the perspectives of individuals with disabilities and their caregivers, highlighting the need for more focused research on these vulnerable populations (Bilik and Akdağ 2023; Ducy and Stough 2021; Eroglu et al. 2024; Kimura 2020; Şahin and Kaya 2025; Yarımkaya and Bakkaloğlu 2024).

Although there is a growing body of literature on disasters and disability, existing studies have largely focused on disaster preparedness, evacuation behaviours, physical impacts, and stakeholder perspectives, while the psychological outcomes experienced by individuals with ID have received limited attention. Recent studies have examined the psychological impact of the Kahramanmaraş earthquake on adolescents (e.g., Kaplan, Alkasaby, et al. 2024; Kaplan, Düken, et al. 2024; Düken, Küçükoğlu, and Kılıçaslan 2024; Düken, Kaplan, and Almazan 2024); however, research specifically focusing on the emotional and behavioural problems of children and youth with ID remains limited.

In this context, addressing the emotional and behavioural needs of children and youth with intellectual disabilities in the aftermath of an earthquake, supporting their participation in social life, developing inclusive emergency preparedness, and ensuring access to mental health services are among the key factors that can promote their healthy development within a more inclusive society. Identifying emotional and behavioural problems observed in children and adolescents with intellectual disabilities in the period following the earthquake is therefore important for contributing to the development of effective interventions and psychological support programs for this population, as well as for guiding future research. It is important to note that the present study does not include pre‐earthquake data; therefore, the findings are limited to describing emotional and behavioural problems observed in the post‐earthquake period and do not allow for causal inferences.

Accordingly, the present study aims to determine the levels of emotional and behavioural problems observed in children and youth with intellectual disabilities in the post‐earthquake period; to examine whether these problems differ according to age and gender; and to evaluate emotional and behavioural difficulties based on findings from the projective drawing test. In this respect, the study is expected to contribute original evidence to the disaster and disability literature by focusing on an underexplored population and outcome domain.

## 2 Methods

### 2.1 Research Model

In this study, an exploratory sequential design from mixed‐method research was used. In the first stage of this design, quantitative data that primarily respond to the research question are collected and analysed. In the second stage, qualitative data are collected and analysed. The researcher uses qualitative results to explain the quantitative results of the first stage (Creswell et al. 2003).

**FIGURE 1:**
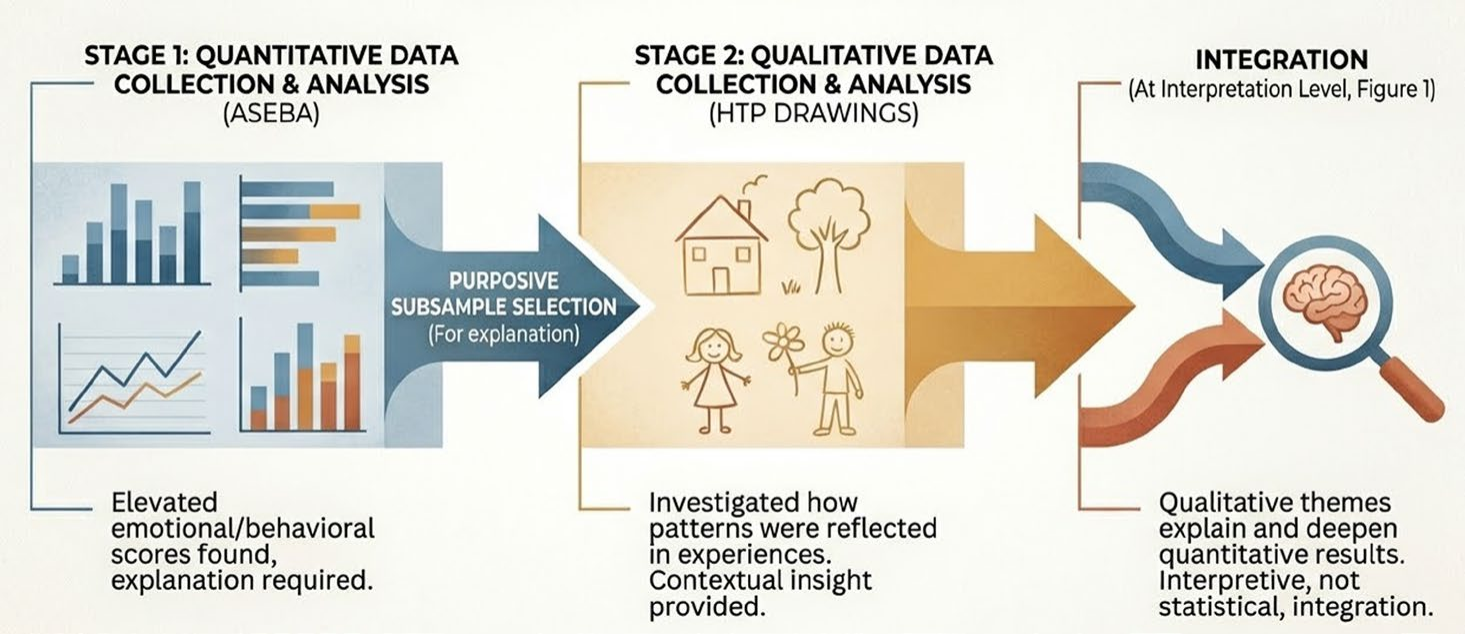
An exploratory sequential design.

This design was implemented in the current study as follows: Quantitative findings obtained from the Achenbach System of Empirically Based Assessment (ASEBA) revealed elevated emotional and behavioural scores in specific domains, which required further explanation. Based on these quantitative results, a purposive subsample was selected for the qualitative phase to explore how these emotional and behavioural patterns were reflected in children's experiences. Qualitative data obtained through the House–Tree–Person (HTP) drawings were used to provide contextual and interpretive insights into the quantitative findings. The integration of quantitative and qualitative data was conducted at the interpretation and conclusion levels, where qualitative themes were used to explain and deepen the understanding of the quantitative results rather than at the statistical analysis level (Figure 1).

### 2.2 Ethical Considerations

Ethical approval was obtained from the relevant institutional ethics committee. Since the study data will be obtained from the teachers and students of the students with ID studying in schools affiliated with the ministry, permission to apply the scale and projective test was obtained from the ministry. Participating teachers and young people voluntarily stated they wanted to participate in the study and signed the participant consent forms. In addition, written consent forms were obtained from the families of children and young people, and permission to participate in the study was obtained.

### 2.3 Participants

#### 2.3.1 Teachers

Participating teachers were selected based on the following criteria: (a) having students with a diagnosis of ID in their class and (b) having worked with these students for at least 6 months prior to the earthquake. Teachers who met the criteria were included in the study. Of the participating teachers, 69.23% were female, and 30.77% were male. 38.46% of the participating teachers were between 20 and 30, while 61.54% were between the ages of 30 and 50. About 69.23% of the teachers have 1–10 years of experience, and 30.77% have 10–20 years of experience. Table 1 presents demographic information about the teachers who participated in the study.

**TABLE 1:** Demographic information about teachers.

Category	*n*	%
Female	9	69.23%
Male	4	30.77%
20–30 age	5	38.46%
30–50 age	8	61.54%
1–10 years experience	9	69.23%
10–20 years experience	4	30.77%
Total	13	100%

#### 2.3.2 Children and Youth

Participants consisted of children and adolescents with intellectual disabilities affected by the February 6 Kahramanmaraş earthquake. Information about the children and adolescents was obtained through interviews with their teachers, who reported observing behaviours such as anxiety, crying, difficulty staying indoors, and frequently leaving the classroom following the earthquake. Teachers also indicated that the children were exposed to earthquake‐related news through the media and to discussions about the earthquake within their households. The study did not differentiate between direct and indirect/secondary exposure to the earthquake, and specific measures of media exposure, displacement, or loss were not directly collected.

Participating children and adolescents were selected using a purposive sampling method with criterion‐based inclusion: (a) having received a diagnosis report for ID from the Department of Paediatric Mental Health Diseases of a university or state hospital, and (b) being between the ages of 6 and 18. Children and adolescents who met these criteria were included in the study. This sampling approach was chosen to ensure that all participants had a confirmed diagnosis of ID and fell within the developmental age range relevant for examining post‐earthquake emotional and behavioural responses.

In the quantitative part of the study, 51.92% of the children who participated in the survey were girls, and 48.08% were boys. Of the participant children (*n* = 52), 11.54% were between the ages of 6 and 12, and 88.46% were between 13 and 18. Table 2 presents the demographic information of the children who participated in the quantitative study.

**TABLE 2:** Demographic information about children and youth.

Category	*N*	%
Gender		
Girl	27	51.92%
Boy	25	48.08%
Ages		
6–12 ages	6	11.54%
13–18 ages	46	88.46%
Educational stage		
Primary school	2	3.85%
Middle school	4	7.69%
High school	46	88.46%
Diagnosis		
ID	52	100%
ID + PD	8	15.38%
ID + VI	1	1.92%
ID + ASD	11	21.15%
ID + HI	2	3.85%
ID + ASD + SLD	2	3.85%
ID + SLD	1	1.92%
Total	52	100%

Abbreviations: ASD, autism spectrum disorders; HI, hearing impairment; PD, physical disability; SLD, speech and language disorder; VI, visual impairment.

In the qualitative part of the study, the sample (*n* = 15) was drawn from the quantitative sample on a voluntary basis. Among the participants who met the inclusion criteria and agreed to participate in the qualitative phase, a purposive selection was applied to ensure relevance to the quantitative findings. The qualitative sample consisted of 15 children and adolescents, 8 girls and 7 boys, aged between 15 and 18.

### 2.4 Data Collection Tools

#### 2.4.1 Demographic Information Form

The researchers developed the Demographic Information Form by reviewing the literature and taking expert opinions. In the form, three questions ask variables such as age, gender, and professional experience of the teacher participants.

#### 2.4.2 ASEBA Teacher Form for Children and Youth Aged 6–18

The ASEBA assesses the emotional and behavioural problems exhibited by children and adolescents in the last 2 months through teacher reports. Validity and reliability studies regarding the adaptation of the scale into Turkish were reported by Erol and Şimşek (2010). The scale is scored as 0, 1, and 2 on a three‐point Likert scale. Three different emotional and behavioural problem scores are obtained from the scale: internalising, externalising, and total problem. Introverted behaviours consist of 33 items, including anxiety/depression, social introversion/depression, and somatic complaints subtests. Introverted behaviours include behaviours that are difficult to observe and evaluate. Externalising behaviours consist of rule‐breaking and aggressive behaviours subtests and are comprised of 32 items. Externalising behaviours refer to clearly and explicitly demonstrated behaviours and include impulsive behaviours with disruptive and destructive effects that are not accepted by social norms. The total problem score consists of internal orientation, external orientation, social problems, thought problems, and attention problems subtests that are not included in these two areas, and the attention problems test is divided into inattention and hyperactivity‐impulsive subtests within itself, and the whole scale contains 120 items. The test–retest reliability of the scale was calculated as 0.88 for the total problem dimension obtained from the entire scale. The internal consistency was found to be 0.89 for internalising, 0.93 for externalising, and 0.96 for the total problem due to Cronbach's alpha coefficients (Erol and Şimşek 2010).

#### 2.4.3 HTP Projective Picture Test

Projective tests, one of the three main testing techniques in psychology, compensate for the limitations of scales (Xiong and Ye 2012). The HTP drawing test was developed by Buck in 1948 and is one of the most widely used projective tests today (Buck 1948b; Guo, Yu, et al. 2023; Guo, Feng, et al. 2023). The HTP is a projective test to assess individuals' personality integration, cognitive maturity, and interpersonal relationships (Oster and Crone 2004, 92). According to a study by the American Psychological Association, the HTP ranks 8th among 102 widely used psychological tests (Camara et al. 2000). The HTP test has a non‐verbal assessment structure. Therefore, the test can conceal the intended purpose and overcome the participants' defence psychology. On the other hand, the HTP is an advantageous assessment tool because the picture is not affected by the participants' culture and can reflect personality traits and potential psychological problems more accurately (Sheng et al. 2019; Smeijsters and Cleven 2006). It is thought that the emotions and psychological states of individuals who make freehand drawings are reflected by symbolic images. ‘House’ refers to the individual's family life and relationships; ‘tree’ refers to interpersonal interactions and ties with the environment; ‘human’ refers to the conscious and unconscious representation of self, personality, relationships with others, and personal‐social attitudes (Groth‐Marnat and Roberts 1998; Hammer 1997). Drawing stimuli can potentially arouse children's emotions and thoughts actively. According to the evaluation results in the Yearbook of Mental Measurements (Dowd 1998; Knoff 1998), the HTP method offers adequate inter‐rater reliability ranging from *α* = 0.70 to *α* = 0.96. In addition, the technique has been used to score abuse in specialised settings quantitatively and has demonstrated validity in distinguishing sexually abused children from non‐abused children.

### 2.5 Data Collection

Following the approval of the ethics committee, visits were made to three special education schools in a province in Türkiye, and information about the study was provided. Teachers and students who met the study criteria were reached. Written informed consent was obtained from parents for their children's participation in the study. During the visits, teachers reported observing a marked increase in anxiety and restlessness among children in the post‐earthquake period, including frequent crying, startle responses, heightened sensitivity to sudden noises, difficulty staying indoors, problems sustaining attention in the classroom, and frequently leaving the classroom. In addition, it was noted that some children exhibited difficulties falling asleep, reluctance to be alone, separation anxiety, and challenges in adapting to daily routines. To collect data using the ASEBA scale, teachers were instructed to read the guidelines of the data collection tools, complete all items fully and impartially, and submit the completed forms to the researchers. Scale forms completed by 13 teachers for 52 students were collected. Forms with incomplete or incorrect responses were excluded from the dataset and analysed by the first researcher.

The classes of the students who were evaluated using the ASEBA scale were visited, and brief information about the HTP test was given. Then, the students were asked whether they would like to participate in the HTP test. Fifteen young people who wanted to participate voluntarily were identified. The HTP projective test was carried out in small groups (4–6 children) with tables and chairs in the classroom environment. The first researcher guided each group. The researcher read the drawing instructions to the students and gave the following instructions: ‘Draw a house. Draw a tree. Draw a person. There is no right or wrong picture of a house, tree or person. We just want to look at your pictures’. The researcher carefully guided each student to complete their drawings with minimal interference from other students and stimuli in the classroom environment. The students were given 15 min for each drawing. The completed drawings of houses, trees, and people were collected in the order of application. Students were also allowed to take their crayons and extra blank paper home. Each drawing was coded with a number, and this number was matched with a list of the participant's name, gender, and age. The researcher did not ask the students additional questions after drawing.

### 2.6 Reliability Analyses

For the current study, Cronbach's alpha coefficients were calculated as 0.88 for internalising, 0.93 for externalising, and 0.96 for the total problem. Since the calculated Cronbach's alpha coefficients are close to the adapted version of the scale, it can be said that the internal consistency level is at the expected level, and it is a reliable measurement tool for this study. For the inter‐rater reliability analyses of the HTP drawing test, a coding scheme was developed based on the qualitative analysis categories proposed by Buck (1948a), and reliability analyses were conducted for the figures' sub‐dimensions. The coding framework and the statistical sub‐dimensions were shared with the researcher and two independent experts from the psychology department. The similarities and differences in the coding of each sub‐dimension were compared across the three independent raters. Miles and Huberman's (1994) reliability formula (Reliability = agreement/(agreement + disagreement) × 100) was used to calculate inter‐rater reliability. The inter‐rater reliability result was 86.20%.

### 2.7 Analysing the Data

ASEBA data were collected from 52 children and youth from three schools affiliated with the Ministry of National Education. Before analysing the research data, the scale forms were first checked, and the scales with eight or more blank items were excluded from the analysis by the scale application directive. Then, coding errors and deficiencies in the data to be analysed were examined, and these errors and deficiencies were eliminated on the scale forms, extreme values were determined, and descriptive data related to the research were checked. The data of the study were analysed using the SPSS 25.0 package program. Kolmogorov Smirnov and Shapiro Wilk tests were applied to test whether the research data were normally distributed, and it was determined that the research data did not show normal distribution. In the study, the Whitney *U* test was used to evaluate the total scores of children and young people from the scale and its sub‐dimensions according to gender and age and whether there was a significant difference between them.

The HTP drawings obtained from 15 participants were analysed by the second researcher. The second researcher is a certified administrator of the HTP projective drawing test and has received training in the analysis of children's drawings. The house, tree, and person figures were categorised according to the sub‐dimensions identified by Buck (1948a), and the drawing features were evaluated as ‘with/without reservations’ or ‘present/absent’. In interpreting the findings, the qualitative analysis approach proposed by Buck (1948a) was followed, and a holistic evaluation was conducted by considering the quality and quantity of details in the drawings, the sequence of drawing elements, emphasised/repeated features, the relationships among the figures, and the overall organisation of the drawings. In line with Buck's (1948a) recommendations, additional/irrelevant details included in the drawings, as well as elements such as the groundline, were also incorporated into the interpretation. Furthermore, marked deviations in detail sequence and patterns of repetition/emphasis were evaluated within the overall organisation of the drawings using a probability‐based interpretive language. First, each drawing was numbered and examined in detail according to the relevant categories. In addition, the analyses conducted by the second researcher were compared with the evaluations carried out independently by two expert psychologists; results were established based on the findings for which consensus was reached, and the reliability of the analysis process was supported through expert review.

## 3 Findings

This study presents findings aimed at determining the levels of emotional and behavioural problems observed in children and youth with intellectual disabilities following the February 6 Kahramanmaraş earthquake, examining whether these problems differ by age and gender, and evaluating emotional and behavioural difficulties based on projective drawing test findings. In this respect, the first two research questions were addressed through results obtained from quantitative data (scale scores and age/gender comparisons), whereas the third research question was evaluated using qualitative findings derived from the HTP projective drawing test.

### 3.1 Quantitative Findings

The overall mean score of emotion and behaviour disorders of children and young people with ID was found to be 33.23. When the sub‐dimensions of the scale were examined, it was determined that the highest mean score was for other problems (18.11), and the mean score of externalising problems (7.63) was followed by internalising problems (7.48). It was found that children with ID had other problems (social, attention, and thought problems) and exhibited aggressive behaviours from externalising problems (Table 3).

**TABLE 3:** Mean and standard deviation of emotion and behaviour disorders scores.

Sub dimensions	*X̄*	SS
Internal orientation problems	7.480769	7.323051
Anxiety and depression	3.326923	3.405423
Social introversion	2.788462	3.488561
Somatic complaints	1.365385	2.457712
External orientation problems	7.634615	10.450685
Disobeying the rules	2.134615	3.559185
Aggressive behaviour	5.500000	7.255829
Other problems	18.115385	16.360706
Social problems	2.634615	2.890341
Attention problems	2.890341	11.218184
Problems of thought	2.211538	3.626355
Total	33.2308	30.18220

Emotional and behavioural disorders of children and youth with ID affected by the earthquake show a significant difference according to gender (*U* = 213.5 *p* < 0.05). Emotional and behavioural disorders of boys (*X̄* = 30.46) were higher than girls (*X̄* = 21.71). In internalisation problems, boys had a higher mean score than girls. However, this difference is not statistically significant (*p* = 0.5). In external orientation problems, boys (*X̄* = 30.34) have a higher mean score than girls (*X̄* = 21.82), and this difference is statistically significant (*p* = 0.03). In other problems, boys (*X̄* = 30.98) had a higher mean score than girls (*X̄* = 21.71), and this difference was statistically significant (*p* = 0.01). The Cohen's d value calculated over the total scores of emotional and behavioural disorders is 0.0562. In this case, the d value of 0.0562 indicates a small effect size. In terms of total scores between genders, the effect of gender was minimal (Table 4).

**TABLE 4:** Mann–Whitney *U* test according to gender variable.

Subscales	Gender	*N*	*X̄*	*U*	*p*
Internal orientation problems	Girl	26	24.80	294.0	0.5
	Boy	25	27.24		
External orientation problems	Girl	26	21.82	216.5	0.03
	Boy	25	30.34		
Other problems	Girl	26	21.21	200.5	0.01
	Boy	25	30.98		
Total	Girl	26	21.71	213.5	0.03
	Boy	25	30.46		

Emotional and behavioural disorders of children and adolescents affected by the earthquake and who have ID do not show a significant difference according to age (*U* = 89; *p* > 0.05).

### 3.2 Qualitative Findings

The drawings of children and youth with ID were generally analysed using seven HTP projective drawing test dimensions. In the analyses, six drawings in the dimension of picture details related to the colours used and their properties, one drawing in the dimension of part‐detail integrity, 13 drawings in the dimension of location‐ground detail, one drawing in the dimension of border lines, and two drawings in the dimension of page centre distance were found to be with reservations in terms of emotional and behavioural disorders. In the dimension of eraser use, all pictures were evaluated as having no reservations regarding emotion and behaviour disorders (Tables 5 and 6).

**TABLE 5:** General picture details for the house–tree–person drawing projective picture test.

Picture	Colours and their features	Details	Part‐detail integrity	Using the eraser	Place floor detail	Edge lines	Page centre distance
1	W/o	W/o	W/o	W/o	W/o	W/o	W/o
2	W/.	W/.	W/.	W/o	W/.	W/.	W/.
3	W/o	W/o	W/o	W/o	W/.	W/o	W/o
4	W/o	W/.	W/o	W/o	W/.	W/o	W/o
5	W/o	W/o	W/o	W/o	W/.	W/o	W/o
6	W/o	W/.	W/o	W/o	W/.	W/o	W/o
7	W/o	W/o	W/o	W/o	W/.	W/o	W/o
8	W/o	W/o	W/o	W/o	W/.	W/o	W/.
9	W/.	W/o	W/o	W/o	W/.	W/o	W/o
10	W/o	W/o	W/o	W/o	W/.	W/o	W/o
11	W/.	W/.	W/o	W/o	W/.	W/o	W/o
12	W/o	W/o	W/o	W/o	W/.	W/o	W/o
13	W/o	W/o	W/o	W/o	W/o	W/o	W/o
14	W/.	W/.	W/o	W/o	W/.	W/o	W/o
15	W/o	W/.	W/o	W/o	W/.	W/o	W/o
Total %	%73.33	%60	%93.33	%100	%13.33	%93.33	%86.67

Abbreviations: W/., with reservations; W/o, without reservations.

**TABLE 6:** Example pictures.

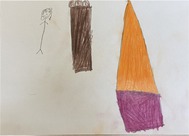 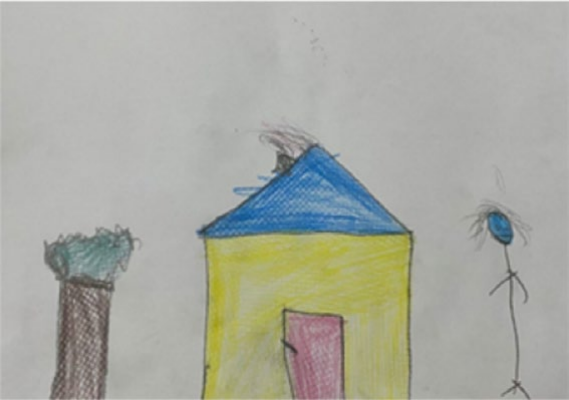 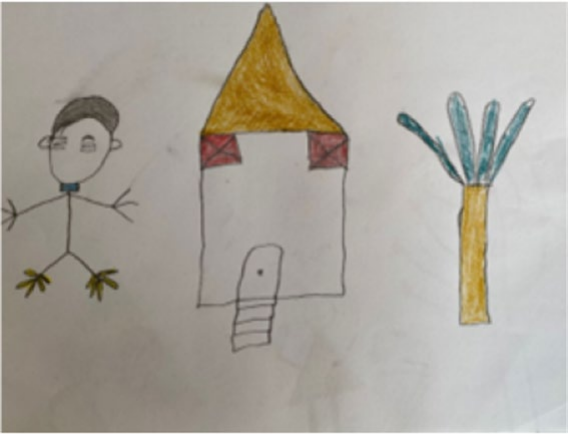

*Note:* Picture: 2, gender: girl, age: 15. Picture: 9, gender: boy, age: 18. Picture: 14, gender: boy, age: 18.

The drawings made by children and young people with ID were analysed using eight dimensions of a house drawing. The analyses determined that there were nine drawings without chimney detail, one without door detail, one without window detail, 12 without stair detail, and eight without other details (house road, attic, etc.). As a result of the analysis, it was determined that the roof and wall details were present in all pictures, but the drawings related to other parts of the house were not included in any picture (Table 7).

**TABLE 7:** House drawing details for the house–tree–person drawing projective picture test.

Picture	Roof	Chimney	Wall	Door	Window	Stairs	Parts of the house	Other details
1	P	P	P	P	P	A	A	P
2	P	P	P	A	P	A	A	A
3	P	A	P	P	P	A	A	P
4	P	P	P	P	P	A	A	A
5	P	A	P	P	P	A	A	P
6	P	P	P	P	P	A	A	P
7	P	P	P	P	P	A	A	P
8	P	P	P	P	P	P	A	P
9	P	P	P	P	A	A	A	A
10	P	A	P	P	P	A	A	A
11	P	A	P	P	P	A	A	A
12	P	P	P	P	P	A	A	A
13	P	P	P	P	P	P	A	P
14	P	A	P	P	P	P	A	A
15	P	A	P	P	P	A	A	A
Total %	%100	%60	%100	%93.33	%93.33	%20	%0	%46.67

Abbreviations: A, absent; P, present.

The drawings made by children and young people with ID were analysed using six dimensions of tree drawings. In the analyses, it was determined that there were singular drawings in 13 drawings and plural drawings in two drawings, the tree was drawn alive in 13 drawings, dried up in two drawings, leaf details were not included in 14 drawings, one drawing did not include leaf details, the tree was medium‐sized in 10 drawings, large in three drawings and tiny in two drawings. When the other information related to the tree drawing was analysed, it was determined that other details (apple, flower, swing, and bird) were included in five drawings, and no details were included in 10 drawings. In all drawings, it was determined that trunk and branch details related to the tree were included (Table 8).

**TABLE 8:** Tree picture details for the house–tree–person projective picture test.

Picture	Single and multiple	Live/dry	Tree trunk/branches	Leafs	Tree size	Other details
1	S	Li	P	A	M	A
2	S	D	P	A	M	A
3	S	Li	P	A	B	P
4	S	Li	P	A	M	A
5	S	Li	P	A	B	P
6	M	Li	P	A	L	P
7	S	Li	P	A	M	A
8	S	Li	P	A	M	P
9	S	Li	P	A	M	A
10	S	Li	P	A	B	P
11	S	D	P	A	M	A
12	M	Li	P	A	L	A
13	S	Li	P	A	M	A
14	S	Li	P	P	M	A
15	S	Li	P	A	M	A
Total %	%100	%100	%100	%6.67	%100	%33.33

Abbreviations: A, absent; B, big; D, dry; L, little; Li, live; M, medium; M, multiple; P, present; S, single.

The drawings made by children and youth with ID were analysed using 15 dimensions of the personal drawing. In the analyses, it was determined that head, eye, and arm details were included in 15 drawings; hair, neck, mouth, and facial expression details were included in 14 drawings; hands and feet were drawn in eight drawings; torso details were included in seven drawings, ears were drawn in five drawings, and other details (dress patterns, buckles, etc.) were drawn in two drawings. As a result of the analysis, it was determined that beard‐moustache and belt details were not drawn in 15 drawings (Table 9).

**TABLE 9:** Person picture details for the house–tree–person projective picture test.

Picture	Head	Hair	Neck	Eye	Mouth	Tooth	Ear	Facial expression	Body	Arm	Hand	Foot	Beard moustache	Belt	Other details
1	P	P	P	P	P	A	A	P	P	P	P	P	A	A	P
2	P	P	P	P	P	P	P	P	P	P	P	P	A	A	A
3	P	P	P	P	P	A	A	P	A	P	A	A	A	A	A
4	P	P	P	P	P	A	P	P	A	P	P	A	A	A	A
5	P	P	A	P	P	A	A	P	P	P	P	P	A	A	A
6	P	P	P	P	P	A	A	P	A	P	A	A	A	A	A
7	P	A	P	P	P	A	P	P	A	P	A	A	A	A	A
8	P	P	P	P	P	A	A	P	P	P	P	P	A	A	A
9	P	P	P	P	P	A	A	P	A	P	A	A	A	A	A
10	P	P	P	P	P	A	P	P	P	P	P	P	A	A	A
11	P	P	P	P	P	A	A	P	A	P	A	A	A	A	A
12	P	P	P	P	P	A	A	P	A	P	A	A	A	A	A
13	P	P	P	P	P	A	A	P	P	P	P	P	A	A	P
14	P	P	P	P	A	A	P	A	A	P	P	P	A	A	A
15	P	P	P	P	P	A	A	P	P	P	A	P	A	A	A
Total %	%100	%93.33	%93.33	%100	%93.33	%0	%33.33	%93.33	%46.67	%100	%53.33	%53.33	%0	%0	%13.33

Abbreviations: A, absent; P, present.

## 4 Discussion

The present study aims to describe the levels of emotional and behavioural problems observed in children and youth with intellectual disabilities in the post‐earthquake period; to examine whether these patterns differ according to age and gender; and to evaluate emotional and behavioural tendencies based on findings from the projective drawing test. The quantitative results indicate that individuals with intellectual disabilities displayed notable emotional and behavioural difficulties in the post‐earthquake period. According to ASEBA results, the observation that children with ID exhibited social, attention, and thought problems corresponds with prior literature reporting increased anxiety and depressive symptoms in children exposed to disasters (Ducy and Stough 2021; Myers 1994; Salloum and Overstreet 2008; Schonfeld and Demaria 2015; Molteno et al. 2001). In particular, boys demonstrated higher levels of rule‐breaking and aggressive behaviour compared to girls, which is consistent with previous literature reporting greater defiance of rules and aggressive behaviour among boys (Baudewijns et al. 2018). This pattern may suggest that boys and young people have a higher tendency to externalise their internal distress, which, when combined with overt behavioural problems, may contribute to increased internal conflict. Recent studies on the February 6, 2023, Kahramanmaraş earthquakes support our findings. One study of 947 adolescents reported high levels of post‐traumatic stress and psychological symptoms, with factors such as time under rubble and familial losses intensifying anxiety, depression, and somatisation (Düken, Küçükoğlu, and Kılıçaslan 2024; Düken, Kaplan, and Almazan 2024). Another study of 704 adolescents highlighted that trauma disrupted mental health and lowered future expectations, underscoring the need for rapid psychosocial support and interventions addressing basic needs (Kaplan, Alkasaby, et al. 2024; Kaplan, Düken, et al. 2024). Together, these studies provide context for understanding emotional and behavioural patterns observed in children and youth in the post‐earthquake period, emphasising the importance of early and comprehensive psychosocial support.

In line with these findings, qualitative research conducted after the 2017 California wildfires demonstrated that parents of children with disabilities reported intense trauma‐related stress responses, grief, and emotional dysregulation, highlighting the profound psychological consequences of disaster‐related disruptions for this vulnerable population (Ducy and Stough 2021). The main stressors identified in the study include disruptions in routines, evacuation barriers, and limited access to disability‐responsive mental health services. These factors may help explain the behavioural and cognitive difficulties observed in our sample, suggesting that both direct and indirect disaster exposure, as well as systemic response gaps, may contribute to adverse outcomes. In this context, it can be stated that the findings may be related not only to direct trauma responses but also to secondary stressors (e.g., media exposure, parental stress, disruptions in access to services) and the disruption of daily routines.

In Türkiye, emerging evidence following the 6 February 2023, Kahramanmaraş earthquakes has consistently highlighted that children and adolescents with developmental disabilities constitute a highly vulnerable group in terms of psychological adjustment, behavioural regulation, and access to continuity of care. In this regard, our quantitative findings indicating elevated social, attention, and thought problems among children and youth with ID are consistent with studies conducted with children with autism spectrum disorder (ASD) living in earthquake‐affected provinces. For instance, Eroğlu and Yakşi (2023) reported that children and adolescents with ASD in Hatay experienced a significant post‐earthquake increase in symptom severity, including hyperactivity, stereotypical behaviours, sleep‐related anxiety, and deterioration in social communication skills, suggesting that disaster‐related stress and disruption of daily routines may exacerbate behavioural and emotional dysregulation in neurodevelopmental conditions. Similarly, Eroglu et al. (2024) emphasised that families of children with ASD experienced persistent challenges in maintaining daily functioning and adaptation after the earthquake, reinforcing the view that environmental instability and loss of routine represent major risk factors for behavioural difficulties. Moreover, research focusing on physical activity participation after the earthquakes demonstrated that ASD‐related behavioural problems (e.g., irritability and increased anger), psychological distress (e.g., separation anxiety), and environmental constraints substantially reduced children's engagement in physical activity, which may further contribute to emotional dysregulation and decreased well‐being (Yarımkaya et al. 2024).

In parallel, qualitative evidence from parents of individuals with ID indicates that families faced multiple barriers during the disaster and recovery period, including limited preparedness, lack of accessible evacuation planning, prolonged insecurity, and insufficient disability‐responsive support services (Yarımkaya and Bakkaloğlu 2024). Likewise, Şahin and Kaya (2025) reported that children with ASD and/or ID displayed increased problem behaviours, fear responses, sleep disturbances, and difficulties associated with disrupted routines and interrupted education, while parents described intense stress, helplessness, and unmet basic needs. Taken together, these Türkiye‐based findings converge with our results by demonstrating that post‐earthquake emotional and behavioural difficulties in children with ID are shaped not only by individual vulnerabilities but also by systemic disruptions such as limited access to specialised services, barriers to safe housing, and discontinuity in education and rehabilitation. Therefore, disability‐inclusive disaster preparedness and coordinated post‐disaster psychosocial support models remain critical priorities to reduce the long‐term emotional and behavioural burden among children and youth with neurodevelopmental disabilities. In the Turkish context, government agencies have played a leading role in addressing the needs of individuals with disabilities following the earthquake. The Ministry of National Education (MNE) has actively developed strategies to ensure educational continuity and provided specialised materials and resources for students with special needs (Özer 2023). Simultaneously, the DEMP spearheaded emergency operations that included vulnerable groups and facilitated social services through collaborations with MNE and local governments (Şensin and Gökmenoğlu 2024). The provision of aid materials specifically designed for the unique requirements of individuals with disabilities is considered a vital step in fostering their social reintegration during the post‐disaster rehabilitation process (Özer 2023).

Moreover, children with ID often exhibit heightened vulnerability to emotional and behavioural disturbances after traumatic events, a pattern corroborated by studies on Hurricane Katrina and other disasters (Lai et al. 2014; Aker and Önder 2025; Başkaya 2025). These findings indicate that pre‐existing cognitive and social vulnerabilities interact with disaster‐related stressors, including housing instability, unmet basic needs, and disruption of daily routines, to exacerbate emotional dysregulation and behavioural problems (Emerson 2003; Üstün 2025; Şahin and Kaya 2025).

This vulnerability underscores the critical role of supportive interventions. Evidence suggests that both children with ID and their caregivers are at risk of experiencing a wide range of emotional and behavioural difficulties following earthquakes and similar disasters (Aslan and Şahinöz 2023; Park et al. 2019; Stough et al. 2017; Valenti et al. 2012). For example, parents reported that their children with ID experienced sleep disturbances, irritability, anxiety, and persistent sadness after the earthquake, alongside adverse impacts on parental mental health (Yarımkaya and Bakkaloğlu 2024). Such findings highlight that disasters represent a serious challenge not only for individuals with disabilities but also for their families (Handicap International 2015). Therefore, it is vital to ensure the provision of post‐disaster psychosocial support services for all individuals. When universal service delivery is not feasible, existing psychosocial support services should be made more accessible for disadvantaged groups (Handicap International 2015). In this context, timely psychosocial support, involvement of special education teachers, and programs promoting self‐compassion and emotional regulation may mitigate the long‐term psychological impact of disasters on children with ID (Yücel et al. 2025; Ducy and Stough 2011; Kimura 2020).

Taken together, the emotional and behavioral consequences of the Kahramanmaraş earthquake for children and youth with ID are consistent with previous disaster research, reinforcing the need for disability‐inclusive disaster preparedness strategies and tailored post‐disaster psychosocial interventions. These findings highlight the interplay between individual vulnerabilities, environmental disruptions, and systemic support gaps in shaping outcomes after catastrophic events.

The qualitative findings of the study revealed that, based on the analyses of the HTP projective drawing test, some children and youth displayed indicators suggestive of emotional and behavioural challenges in the post‐earthquake period, as reflected in HTP projective drawings, particularly in indicators such as colour use, location/ground details, and page placement; however, eraser use did not indicate any risk across all drawings. In addition, while the basic elements of the house, tree, and person figures were generally included in the drawings, notable omissions were observed in details such as the chimney, stairs, leaves, hands and feet, and the torso. The HTP drawing test provides essential data in terms of reflecting children's feelings and thoughts with symbolic images. It is widely accepted in the literature that HTP is an effective tool for assessing personality integration, cognitive maturity, and interpersonal connections (Oster and Crone 2004). In particular, the strong links in individuals with psychiatric problems such as depression and anxiety (Li et al. 2014; Sheng et al. 2019) and its role in the assessment of psychological effects after natural disasters (Roysircar et al. 2019; Wang et al. 2010) reveal the importance of HTP. In the study conducted by Wang et al. (2010), HTP drawings of children who were directly affected by the earthquake that occurred in Wenchuan, China, in 2008 were analysed, and it was determined that these drawings reflected traumatic experiences. This study evaluated the drawings of broken house walls, bloody tree trunks, and trees with pruned dry branches as negative reflections of children's psychological conditions. In addition, the lack of a face in human drawings and the presence of details such as huge teeth bear traces of aggression and negative self‐concept. Similarly, Nuttman‐Shwartz et al. (2010) analysed the HTP drawings of Jewish children aged 7–9 who were forcibly displaced from the Gaza Strip in Palestine and observed that the trauma indicators of these children included a weak house structure, superficial drawings, and threatening elements. Abandoning the house and broken tree trunks indicate migration threatened the children's lives.

In a meta‐analysis conducted by Guo, Yu, et al. (2023) and Guo, Feng, et al. (2023), 30 studies in which the HTP test was applied to participants with different diagnoses such as autism, cognitive disability, depression, schizophrenia, post‐traumatic stress disorder, and anxiety disorder were examined. According to the research results, the drawings had important predictive features in identifying mental disorders. Among these drawings, the absence of windows, loss of facial features, inappropriate body proportions, and tiny houses and trees were found. In this context, it is thought that the data provided by the HTP test offer a critical opportunity to understand the inner world of children with ID (Roysircar et al. 2019; Wang et al. 2010). The evaluations made through drawings directly reflect children's emotional states and social interactions (Buck 1948a).

House drawings can help to understand the individual's family structure, self‐perception, and interpersonal relationships (Zhang 2010). Studies show that the absence of some elements in drawings gives essential clues about the individual's inner world (Johnson and Johnson 1971). For example, the lack of doors and windows may reflect the individual's reluctance to contact the outside world and their tendencies towards social isolation. According to Chen (2015) study, it was observed that after schizophrenia patients received treatment, doors and windows increased significantly in their drawings. This finding suggests that the need for social interaction was restored during the treatment process. The house's lack of interior details indicates a physical environment and a lack of emotional support (Johnson and Johnson 1971). This situation can be considered a reflection of children's safety concerns and feelings of social withdrawal, especially after traumatic events such as earthquakes. In addition, drawing a smoking chimney in a house symbolises restlessness and anxiety within the family, which may be an important indicator of mental disorders (Chen 2015). In this context, the excessive details in the house drawing may express the individual's concerns about their family and their significant anxiety level.

In‐person and tree drawings, disproportionate and incomplete body details, faces without emotional expression, and singular and dry tree images were observed. Such patterns may reflect the children's emotional state and challenges in social expression in the post‐earthquake period without implying direct causation, have difficulty expressing their emotions, and feel lonely in social relationships. Tree images reflect the relationship between the individual's inner feelings and the external world and represent the emotional experiences of growing up (Cai et al. 2012). Essential features of tree drawings include smallness, size, and dryness. Cut or dry trees symbolise emotional indifference and loss of will to live (Fukunishi et al. 2002). Hui (2014) found dry trees only in depressed individuals. While large trees represent vitality, tiny trees reflect loneliness and lack of self‐esteem. In human drawings, the absence of elements, facial features, or limbs in the figure indicates that the individual's self‐awareness is weakened (Xie and Ye 1994). In addition, the expression of negative emotions is observed more frequently in individuals with mental disorders (Machover 1949). Therefore, HTP test applications are essential to determine these individuals' psychosocial needs and develop appropriate support strategies. All these findings reveal the necessity of special intervention programs to strengthen the mood and sense of security of children and youth with ID after disasters. Countries with many active fault lines, such as Türkiye, should develop plans to address observed emotional and behavioural patterns among vulnerable individuals, such as children and youth with ID, to support their well‐being in post‐disaster contexts before devastating disasters such as earthquakes.

## 5 Limitations

Despite its contributions to understanding the emotional and behavioural difficulties experienced by children and youth with intellectual disabilities following the earthquake, the present study has several limitations. First, the sample size was limited, which restricts the generalisability of the findings; therefore, future studies should target larger populations. In addition, the findings of this study should be interpreted within the Turkish context, as cultural, educational, and service‐related factors specific to Türkiye may have influenced the experiences and responses of the participants; therefore, caution is needed when generalising the results to other countries or settings. In addition, this study does not distinguish between direct and indirect exposure to the earthquake, which limits the interpretation of the findings with respect to specific exposure pathways. Second, the scoring and interpretation of the HTP drawing test are not standardised, and the drawing features selected by the researchers may involve subjective judgement, making it difficult to compare findings across studies (Cai et al. 2012; Chen and Yan 2022). However, given that the HTP test was used to compensate for limitations of standardised scales, it may still provide supportive data that complements and strengthens the overall findings (Xiong and Ye 2012). Finally, the study did not include pre‐earthquake measurements, which limits the ability to compare changes over time and prevents causal interpretations regarding the impact of the earthquake.

## 6 Conclusion

When the quantitative and qualitative findings are considered together, overall, it is evident that emotional and behavioural difficulties observed in the post‐earthquake period among children and youth with intellectual disabilities. It is important to note that the present study does not include pre‐earthquake data; therefore, the findings describe emotional and behavioural difficulties observed in the post‐earthquake period and do not allow for causal inferences. In general, the quantitative findings showed that other problems (social, attention, and thought problems) had the highest mean scores, followed by externalising and internalising problems. Consistent with this pattern, the HTP drawings revealed several features that may be associated with socio‐emotional distress, attentional/organisational difficulties, and reduced emotional expression. In particular, the limited use of contextual elements (e.g., place–ground details and page placement), restricted elaboration of drawings, and the omission of key structural details in house, tree, and person drawings (e.g., chimney, stairs, leaves, hands/ft, torso) may reflect difficulties in planning, attention regulation, and interpersonal engagement. Therefore, the qualitative drawing‐based findings are consistent with the quantitative results, providing complementary descriptive evidence, providing complementary evidence that earthquake‐affected children and youth with intellectual disabilities experienced pronounced difficulties, particularly in the social–cognitive and attention‐related ‘other problems’ domain reported by teachers. In this respect, the qualitative findings support the quantitative evidence by helping to interpret the attention/social functioning difficulties and externalising behavioural problems identified through standardised measures, highlighting that post‐earthquake emotional and behavioural difficulties can be observed not only through scale scores but also through children's symbolic expressions.

## 7 Recommendations: Implications for Practice and Future Research

Addressing the emotional difficulties experienced by earthquake survivors should not be limited to individual‐focused interventions; rather, it should be grounded in a holistic framework in which teachers and families are supported and actively involved in educational processes. In this respect, a school‐based and structured ‘Stress Coping Methods Training’ program can be developed and implemented by teachers to strengthen the coping skills and psychological resilience of children with ID in the face of and following stressors such as earthquakes. For example, Chang et al. (2023) emphasise the importance of considering each child's individual capacities and support needs, providing additional guidance and personalised strategies for children who may require extra support in stressful situations. Accordingly, the program may include brief breathing exercises or individualised relaxation techniques tailored to each child's needs. The program may also include activities aimed at enhancing children's psychological resilience, such as emotional awareness exercises, creating safe spaces, re‐establishing daily routines, fostering social support and communication skills, and creative expression methods such as drawing, play, and storytelling. Rofiah et al. (2024) illustrate that, within a school‐based inclusive disaster risk reduction framework, teachers can implement practical strategies such as identifying children with special needs, ensuring accessibility, promoting meaningful participation, and fostering collaboration and support networks, thereby increasing program effectiveness. In this context, classroom activities can be delivered using visual supports, concrete materials, and short, frequent repetitions. Such interventions have the potential not only to reduce children's post‐earthquake emotional distress but also to support behavioural regulation skills, thereby improving classroom adjustment and participation.

In addition to these recommendations, low‐cost, easily implementable, and rapidly applicable practical strategies should be developed for use in school and home settings. In this context, teachers may use daily emotional check‐in routines (e.g., emotion cards, visual emotion scales, brief sharing activities) to support non‐verbal emotional expression in children with ID; visual daily schedules and predictable classroom routines may help reduce post‐disaster uncertainty and support attention and emotional regulation. Classrooms may include calming spaces/safe corners to support self‐regulation during emotional overload, and brief movement, breathing, and grounding activities should be integrated into daily routines. Trauma‐informed communication guidelines for teachers and caregivers (e.g., use of simple and concrete language, emotional validation, consistent adult responses) should be developed; family‐oriented home‐based guidance materials should support the creation of predictable home environments and emotional expression opportunities. At the school level, individualised support plans should be developed for children with pronounced emotional and behavioural difficulties, and multidisciplinary collaboration among teachers, families, and mental health professionals should be strengthened to ensure continuity of emotional support.

For future research, it is recommended that longitudinal studies be conducted with larger samples across different geographical regions in order to examine the long‐term psychological status of children with intellectual disabilities following disasters. In addition, considering the emotional and behavioural impacts of large‐scale traumatic events on children, such as the recent Russia–Ukraine war and the Israel–Gaza conflict, future studies should be designed to include not only disaster‐affected children (with or without special needs) but also war‐affected children. When assessing traumatic experiences, it is important to take into account that children may have limited verbal expression skills; therefore, increasing research that employs non‐verbal assessment methods, such as projective drawing tests, may contribute to a more comprehensive understanding of children's inner experiences.

## Author Contributions

Hatice Ulu Aydın developed the study, conducted the data collection and statistical analyses, and drafted the manuscript. Emine Eratay conceptualised the research framework, oversaw the methodology and analyses, and performed critical revisions of the manuscript.

## Funding

The authors have nothing to report.

## Disclosure

Permission to reproduce material from other sources: No material from other sources that requires permission was reproduced in this manuscript. All scales and assessment tools used in the study are either utilised within the scope of academic licensing or are in the public domain for research purposes.

## Ethics Statement

The study was conducted in accordance with the requirements of the University of … Human Research Ethics Committee (2023/175).

## Consent

Informed consent was obtained from the legal guardians of all individual participants included in the study. Additionally, institutional permission was granted by the relevant educational authorities. The participants were informed about the study's purpose, and their anonymity was strictly maintained throughout the research process.

## Conflicts of Interest

The authors declare no conflicts of interest.

## Data Availability

The data that support the findings of this study are available on request from the corresponding author. The data are not publicly available due to privacy or ethical restrictions.
